# Effective Use of Mepolizumab in a Rare Case of Eosinophilic Granulomatosis With Polyangiitis Complicated by Pulmonary Tuberculosis

**DOI:** 10.7759/cureus.96102

**Published:** 2025-11-04

**Authors:** Yoshihiro Okada, Makiko Kumamoto, Shigeo Muro, Shinji Tamaki

**Affiliations:** 1 Department of Respiratory Medicine, Saiseikai Suita Hospital, Suita, JPN; 2 Department of Internal Medicine, National Hospital Organization Nara Medical Center, Nara, JPN; 3 Department of Respiratory Medicine, Nara Medical University, Kashihara, JPN

**Keywords:** anti-eosinophil-driving-cytokine agents, eosinophilic granulomatosis with polyangiitis, eosinophilic inflammation, mepolizumab, pulmonary tuberculosis

## Abstract

A 58-year-old woman was referred to our institution for infiltration shadows in the left upper lobe and a positive polymerase chain reaction for *Mycobacterium tuberculosis *(*Mtb*). She had worsening pain and sensory abnormalities in both lower legs. Red skin rashes were observed on both lower legs, which had resolved at the time of referral to our institution. She received oral prednisolone (PSL) monotherapy (10 mg/day) for these symptoms from her previous physician. She had a history of asthma and chronic rhinosinusitis. The nerve conduction studies and blood tests revealed mononeuritis multiplex and abnormally elevated peripheral eosinophil counts. She was diagnosed with eosinophilic granulomatosis with polyangiitis (EGPA) complicated by pulmonary tuberculosis (TB). The PSL dose was increased from 10 mg/day to 50 mg/day (1 mg/kg/day), and anti-TB treatment was initiated. Mepolizumab, a monoclonal antibody targeting interleukin-5, was also administered. Peripheral eosinophil counts were decreased, and her pain and sensory abnormalities were improved. Mepolizumab inhibited eosinophilic inflammation without compromising predominant immune function against *Mtb*. In the present case, mepolizumab was effective in treating EGPA and preventing pulmonary TB exacerbation. This is a valuable report suggesting the crucial role of mepolizumab in combined cases of both conditions.

## Introduction

Eosinophilic granulomatosis with polyangiitis (EGPA) exhibits features of both anti-neutrophil cytoplasmic antibody (ANCA)-associated vasculitis (AAV) and eosinophilic disorder [1]. Tuberculosis (TB) is a life-threatening infection caused by *Mycobacterium tuberculosis* (*Mtb*) [2]. The coexistence of the two diseases is rare. High-dose glucocorticoids and immunosuppressants are used in severe EGPA [3]; however, these medications induce immune dysfunction against pathogens. Thus, treating EGPA complicated by TB is challenging. Mepolizumab, an anti-interleukin-5 monoclonal antibody, has recently garnered attention for its effectiveness in EGPA [4]. Here, we report a rare case of EGPA complicated by pulmonary TB. In the present case, mepolizumab played a crucial role in treating both conditions.

## Case presentation

**Figure 1:**
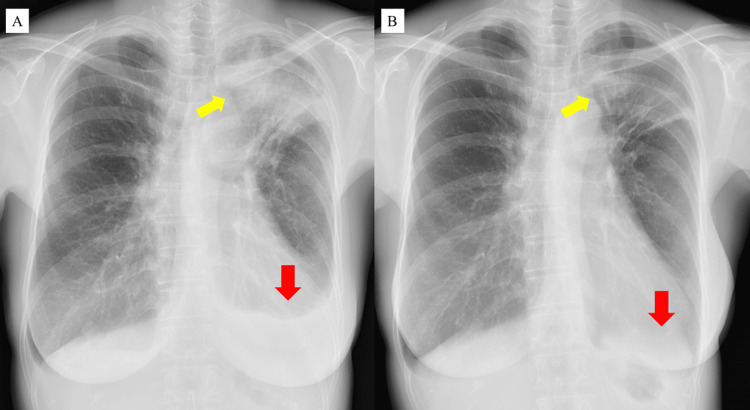
Chest radiography before and after the first treatment Chest radiography shows infiltration shadows in the left upper lung and left pleural effusion (yellow and red arrow) (A). Following the administration of oral prednisolone (10 mg/day) and levofloxacin hydrate (500 mg/day) (after five days), the infiltration shadows improved, and the left pleural effusion had disappeared (B)

**Figure 2:**
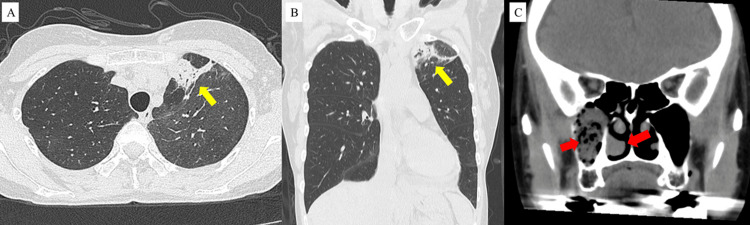
Chest and paranasal sinuses computed tomography Chest computed tomography (CT) shows a consolidation without multiple nodular and cavitary lesions in the left upper lobe (yellow arrow) (A, B). Paranasal sinuses CT shows occupying lesions in the right maxillary sinus and mucosal thickening of the middle turbinate and inferior turbinate on the right side (red arrow) (C)

A 58-year-old Japanese woman with a history of asthma and chronic rhinosinusitis consulted a local hospital for worsening pain and sensory abnormalities in both lower legs that had appeared two weeks earlier. She had been treated with inhaled indacaterol-mometasone (300 µg/day and 160 µg/day) and oral montelukast (10 mg/day), and symptoms of the two diseases had been stable without the use of systemic steroids. At the first consultation, red skin rashes were observed on both lower legs. Wheezing, cough, dyspnea, loss of smell, fever, or weight loss were not observed. A blood test revealed abnormally elevated peripheral eosinophil counts, and chest radiography showed an infiltration shadow in the left upper lung and left pleural effusion (Figure 1A). The involvement of eosinophilic inflammation and infection was suspected. Betamethasone sodium phosphate (4 mg) was injected, and oral prednisolone (PSL) (10 mg/day) and levofloxacin hydrate (500 mg/day) were also administered. After five days, the infiltrative shadow was improved, and the left pleural effusion disappeared on chest radiography (Figure 1B); however, the pain and sensory abnormalities in both lower legs persisted. She was referred to another local hospital for more detailed examinations, including computed tomography (CT) and sputum microbiological tests. Chest CT revealed consolidation in the left upper lobe without multiple nodular or cavitary lesions (Figures 2A, 2B). Paranasal sinuses CT showed mucosal thickening and solid lesions on both sides. In particular, occupying lesions in the right maxillary sinus and mucosal thickening of the right middle and inferior turbinate were prominent (Figure 2C). Sputum microbiological tests revealed a positive polymerase chain reaction for *Mtb *with a negative smear for acid-fast bacilli. Pulmonary TB was suspected, and she was referred to our institution one month after the onset of symptoms.

**Figure 3:**
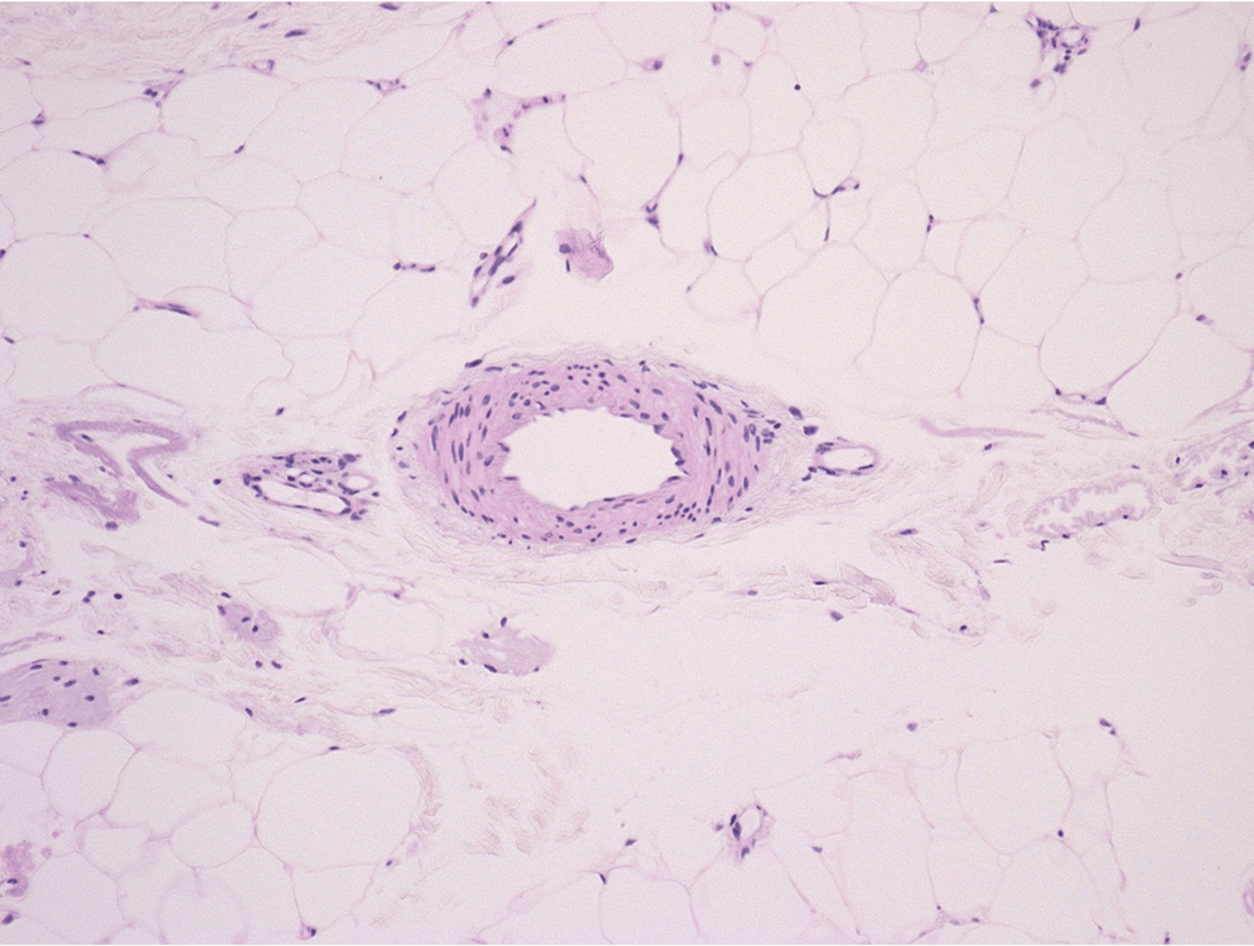
Histological findings of skin biopsy Skin biopsy of the brown pigmentation on the left lower leg, with hematoxylin and eosin staining, reveals no vasculitis, extravascular granuloma, or eosinophilic infiltration

At the time of referral, her pain and sensory abnormalities in both lower legs had worsened, and she even needed a walking cane. A mild cough was observed; however, weight loss, fever, or hemoptysis was not observed. Nerve conduction studies revealed axonal degeneration, suggesting mononeuritis multiplex in both lower legs. A blood test revealed abnormally elevated peripheral eosinophil counts, increased total immunoglobulin E (IgE) levels, and a positive rheumatoid factor. Myeloperoxidase- and proteinase 3-ANCA were negative (Table 1). A skin biopsy was performed on her left lower leg; however, the red skin rashes had already disappeared, and brown pigmentation was observed. The histological findings suggested no vasculitis, extravascular granuloma, or eosinophilic infiltration (Figure 3). Based on the American College of Rheumatology classification criteria in 1990 for EGPA [5] and the criteria proposed by Lanham et al. [6], she was diagnosed with EGPA. In addition, she was diagnosed with pulmonary TB based on microbiological tests. The PSL dosage was increased to 50 mg/day, and four anti-TB drugs (isoniazid, rifabutin, ethambutol, and pyrazinamide) were administered. Rifabutin was preferred to rifampicin owing to its interaction with glucocorticoids. Similarly, pregabalin, loxoprofen sodium hydrate, and tramadol were administered to alleviate the pain. After initiating these therapies, the pain was slightly improved; however, the range of sensory abnormalities in the lower legs extended. Thus, the PSL monotherapy was considered insufficient, prompting the use of mepolizumab to treat EGPA. Subsequently, three days after administering mepolizumab, the peripheral eosinophil counts had decreased to within the normal range. The severity and range of the sensory disorder also gradually improved, and she walked without a cane. Mepolizumab (300 mg) was injected every four weeks, and oral PSL is being tapered to a maintenance dose of 5 mg/day. Regarding pulmonary TB, all culture tests were negative, and the presence of drug resistance was unclear. Thus, the abovementioned four anti-TB drugs are continued.

**Table 1:** Results of blood test at the time of referral to our institution ANCA: anti-neutrophil cytoplasmic antibody; ALT: alanine aminotransferase; AST: aspartate aminotransferase; MPO: myeloperoxidase; PR3: proteinase 3

Variables	Results	Reference range
Complete blood cell count		
White blood cell counts, /µL	17,900	4,000-9,000
Neutrophil, (%)	41.4	45-70
Lymphocyte, (%)	3.1	30-45
Eosinophil, (%)	54.2	2.0-10
Absolute eosinophil conuts, /µL	9,700	40-400
Red blood cell counts, 10^4^/µL	399	380-510
Hemoglobin, g/dL	12	12-16.5
Platelet counts, 10^4^/µL	36.1	35-45
Serum chemistry		
Total protein, g/dL	6.9	6.7-8.3
Albumin, g/dL	3.6	4.0-5.0
Total bilirubin, mg/dL	0.34	0.3-1.2
AST, U/L	28	13-33
ALT, U/L	18	6.0-27
Creatinine, mg/dL	0.5	0.4-0.7
Sodium, mEq/L	139	138-146
C-reactive protein, mg/dL	1.18	<0.3
Immunoserological test		
Total immunoglobulin-E, IU/mL	1,070	<170
Antinuclear antibody	Negative	Negative
MPO-ANCA	Negative	Negative
PR3-ANCA	Negative	Negative

**Figure 4:**
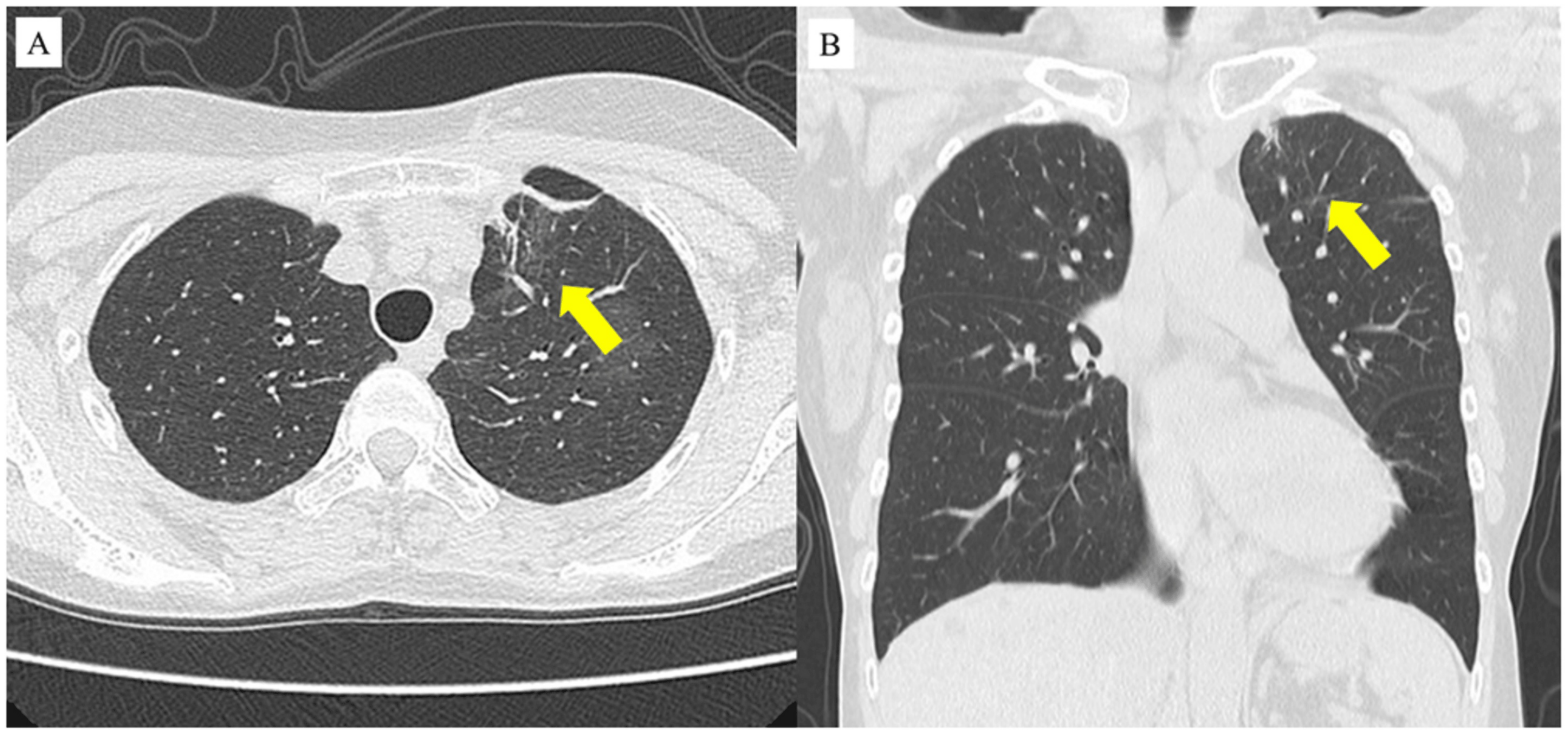
Chest computed tomography after initiating treatment of EGPA and TB EGPA: eosinophilic granulomatosis with polyangiitis; TB: tuberculosis Chest computed tomography shows a reduced area of consolidation and newly emerged scarring shadows eight weeks after initiating treatment for EGPA and pulmonary TB (yellow arrow) (A, B)

**Figure 5:**
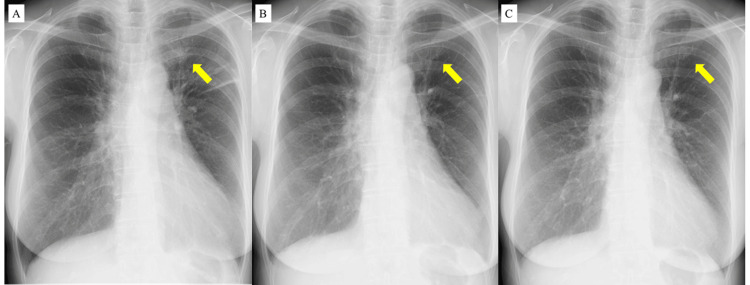
Changes of chest radiography in the clinical course TB: tuberculosis Chest radiography at the time of referral to our institution shows an infiltrative shadow in the left upper lung (yellow arrow) (A). Following the initiation of anti-TB treatment and increased dosage of oral prednisolone, the infiltrative shadow had improved. The infiltrative shadow disappeared at eight weeks after the treatment (B), and no new abnormal shadows appeared until 29 weeks (C)

**Figure 6:**
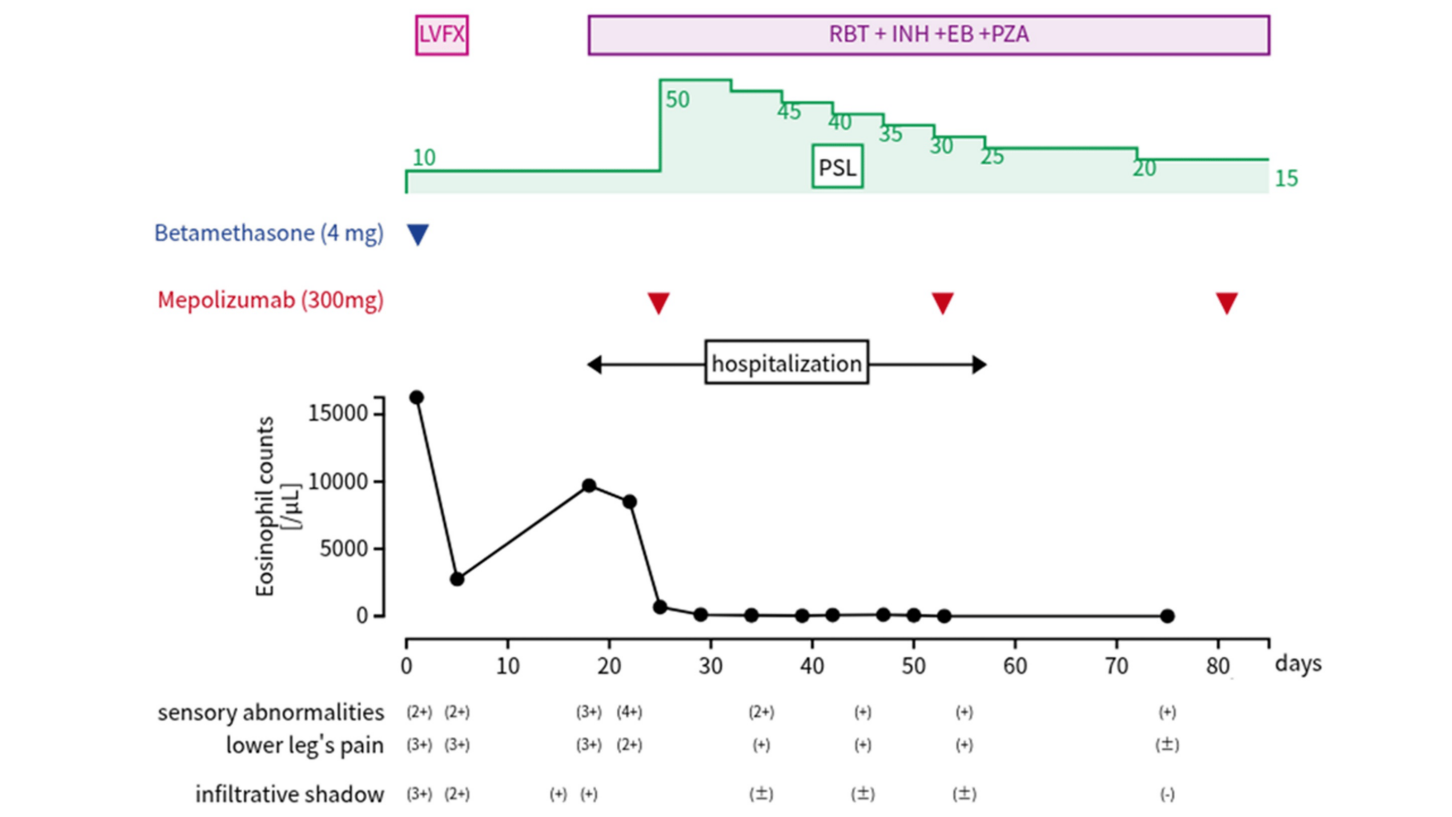
Timeline diagram of the overall clinical course LVFX: levofloxacin; RBT: rifabutin; INH: isoniazid; EB: ethambutol; PZA: pyrazinamide; PSL: prednisolone

Furthermore, eight weeks after initiating the treatment, the consolidation in the left upper lobe had improved with scarring shadows (Figures 4A, 4B). Symptoms or findings of blood tests suggestive of a relapse of EGPA and pulmonary TB were not observed at the last consultation. Radiographically, the improvement of the left upper shadow has not recurred (Figures 5A-5C). The main clinical course is shown in Figure 6. Preceding asthma and chronic sinusitis had not been exacerbated before and after the diagnosis of EGPA.

## Discussion

The detailed mechanism of EGPA remains unclear. The involvement of systemic inflammation induced by eosinophils and ANCA is suspected [1], and negative ANCA cases have been reported [7]. The diagnostic criteria for EGPA have been developed. In daily clinical practice, the ACR 1990 classification criteria and the diagnostic criteria proposed by Lanham et al. are common [5,6]. Although no histological findings suggesting vasculitis were observed in the skin biopsy, the symptoms, clinical courses, and laboratory data met the two criteria without histological findings. Additionally, the microbiological test suggested pulmonary TB complications. Finally, we diagnosed her with a combined case of EGPA and pulmonary TB. Regarding the infiltrative shadow and pleural effusion in the initial consultation, we speculated the involvement of eosinophilic pneumonia based on improvement with five days of systemic steroids and the trend in peripheral eosinophil counts; however, TB lesions were possible. We should have performed bronchoscopy as well as histological and microbiological evaluations. Additionally, screening tests for *Mtb *should have been performed before the administration of levofloxacin. Monotherapy with fluoroquinolones can lead to a refractory infection due to acquired resistance and a delay in the diagnosis of TB [8-10]. We should exercise caution in selecting antibiotics for patients with pneumonia where the involvement of TB cannot be ruled out.

The combined treatment with high-dose corticosteroids and immunosuppressants was recommended in severe EGPA; however, the lengthy use of these drugs induces diverse adverse events, such as compromised immune function, and rapid tapering of steroids causes EGPA relapse [3]. Thus, effective additional treatment methods have been developed, and anti-eosinophil-driving-cytokine agents have garnered attention in the treatment of eosinophilic disorders. In EGPA, Wechsler et al. reported that mepolizumab, an anti-interleukin-5 monoclonal antibody, effectively reduced glucocorticoid dose, achieved remission, and maintained remission, and that no significant induced deadly adverse events were observed in the group with mepolizumab compared with that in the placebo group [4].

The present case involves the coexistence of EGPA and pulmonary TB, a rare phenomenon. Thus, studies and reports on those cases are lacking, and the association between EGPA and TB remains unclear. Lai et al. also reported a case of EGPA combined with pulmonary TB. They treated the patient with PSL monotherapy and anti-TB drugs and successfully controlled both diseases without immunosuppressants. The significance of preserving anti-TB immunity was emphasized [11]. We treated the patient with PSL and mepolizumab in the present case. Therefore, remission and a quick tapering PSL dose without EGPA relapse were achieved. In addition, pulmonary TB was not exacerbated in the clinical course.

TB is an infection caused by *Mtb*, where cytokines, such as interferon-γ, produced by T helper 1 (Th1) cells, are predominant in anti-TB immunity. Th1 cells participate in the activation of macrophages engulfing *Mtb*, as well as in the formation and maintenance of granuloma [12]. In other words, Th1 cell predominant inflammation plays a crucial role in anti-TB immunity. T helper 2 (Th2) cell-predominant inflammation is associated with parasitic infection or allergic diseases. Cross-regulation during Th1 and Th2 cell differentiation has been demonstrated: Th1 and Th2 cells inhibit each other’s differentiation via cytokines [13,14]. Thus, their predominant inflammation cannot co-occur. However, several case reports regarding eosinophilic disorders suggestive of Th2 cell-predominant inflammation in patients with TB have been reported [11,15,16]. In addition, Bohrer et al. reported that eosinophils were enriched in the rim area of the TB lesions in vivo. They hypothesized that eosinophils accumulated in the TB lesions from peripheral blood and play a nondirect protective role against *Mtb *[17]. Th2 cytokines activate and recruit eosinophils to the lungs [18]. Ohrui et al. reported that pretreatment patients had higher baseline serum IgE levels than healthy individuals, suggesting that the initiation of anti-TB treatment decreased serum total IgE levels. Th2 cells participate in the elevation of serum total IgE levels [19]. These reports and studies suggest the roles of Th2 cells in anti-TB immunity. Thus, although the stage is unclear, *Mtb *infection can cause Th2 cell-predominant inflammation in targeted organs, which may induce systemic eosinophilic disorders.

In the present case, pulmonary TB and EGPA were diagnosed simultaneously; however, the preceding condition remains unclear. Preceding EGPA involving Th2 cell-predominant inflammation may suppress Th1 cell-predominant inflammation necessary for anti-TB immunity, triggering the onset of pulmonary TB. In contrast, as mentioned above, *Mtb* infection itself may induce Th2 cell-predominant inflammation. Considering that the onset of TB can be involved in activating Th2 cells in targeted organs and causing systemic eosinophilic disorders, preceding pulmonary TB may have caused eosinophilic inflammation in the lungs, potentially triggering EGPA.

## Conclusions

A combined case of EGPA and pulmonary TB is rare. In treating the two conditions, physicians should control severe systemic inflammation caused by eosinophils and ANCA while preserving the immune function against *Mtb*. Mepolizumab, which selectively suppresses eosinophilic inflammation, is effective for preventing the lengthy use of high-dose glucocorticoids and immunosuppressants, which induce compromised immune function and cause *Mtb *infection. However, it is essential to consider the possibility that eosinophils participate in the immune response to *Mtb* and that mepolizumab affects anti-TB immunity. Therefore, careful monitoring is necessary to ensure that TB does not worsen during treatment with mepolizumab.
